# Evaluation of Persistence of Resistant Variants with Ultra-Deep Pyrosequencing in Chronic Hepatitis C Patients Treated with Telaprevir

**DOI:** 10.1371/journal.pone.0041191

**Published:** 2012-07-27

**Authors:** Xiomara V. Thomas, Joep de Bruijne, James C. Sullivan, Tara L. Kieffer, Cynthia K. Y. Ho, Sjoerd P. Rebers, Michel de Vries, Hendrik W. Reesink, Christine J. Weegink, Richard Molenkamp, Janke Schinkel

**Affiliations:** 1 Academic Medical Center, Department of Medical Microbiology, Section of Clinical Virology, Amsterdam, The Netherlands; 2 Academic Medical Center, Department of Gastroenterology and Hepatology, Amsterdam, The Netherlands; 3 Vertex Pharmaceuticals Incorporated, Cambridge, Massachusetts, United States of America; 4 Academic Medical Center, Department of Medical Microbiology, Section of Human Retro Virology, Amsterdam, The Netherlands; University of Modena & Reggio Emilia, Italy

## Abstract

**Background & Aims:**

Telaprevir, a hepatitis C virus NS3/4A protease inhibitor has significantly improved sustained viral response rates when given in combination with pegylated interferon alfa-2a and ribavirin, compared with current standard of care in hepatitis C virus genotype 1 infected patients. In patients with a failed sustained response, the emergence of drug-resistant variants during treatment has been reported. It is unclear to what extent these variants persist in untreated patients. The aim of this study was to assess using ultra-deep pyrosequencing, whether after 4 years follow-up, the frequency of resistant variants is increased compared to pre-treatment frequencies following 14 days of telaprevir treatment.

**Methods:**

Fifteen patients from 2 previous telaprevir phase 1 clinical studies (VX04-950-101 and VX05-950-103) were included. These patients all received telaprevir monotherapy for 14 days, and 2 patients subsequently received standard of care. Variants at previously well-characterized NS3 protease positions V36, T54, R155 and A156 were assessed at baseline and after a follow-up of 4±1.2 years by ultra-deep pyrosequencing. The prevalence of resistant variants at follow-up was compared to baseline.

**Results:**

Resistance associated mutations were detectable at low frequency at baseline. In general, prevalence of resistance mutations at follow-up was not increased compared to baseline. Only one patient had a small, but statistically significant, increase in the number of V36M and T54S variants 4 years after telaprevir-dosing.

**Conclusion:**

In patients treated for 14 days with telaprevir monotherapy, ultra-deep pyrosequencing indicates that long-term persistence of resistant variants is rare.

## Introduction

Worldwide, an estimated 170 million people are chronically infected with hepatitis C virus (HCV) [1]. Chronic hepatitis C is a major cause of liver cirrhosis and hepatocellular carcinoma. HCV-related end-stage liver disease is now the main indication for liver transplantation in North America and Western Europe [2]. The current standard of care, pegylated interferon-α-2a/b (PEG-IFN) combined with ribavirin (RBV), has limited efficacy and causes significant side effects. In patients infected with HCV genotype 1, the most prevalent genotype in developed countries, treatment for 48 weeks results in rates of sustained virologic response (SVR) of only 40-50%.

Efforts to improve patients’ outcomes have resulted in the development of direct-acting antiviral agents (DAAs) such as non-structural protein 3/4A (NS3/4A) serine protease inhibitors. The NS3/4A protease mediates the cleavage of the HCV polyprotein into functional viral proteins essential for viral replication [3]. NS3/4A serine protease inhibitors block this NS3/4A protease-dependent cleavage [4]–[6].

Two of those protease inhibitors, telaprevir and boceprevir, are now licensed in several countries for clinical use in combination with PEG-IFN and RBV, after extensive preclinical and clinical evaluation [7], [8].

Telaprevir (TVR) is a selective, reversible, orally bio-available NS3/4A protease inhibitor that has demonstrated potent antiviral activity in patients infected with HCV genotype 1 [9], [10]. Phase 3 clinical studies investigating TVR, PEG-IFN and RBV combination therapy demonstrated significant improvement of SVR rates compared to standard treatment in both treatment-naive and prior treatment-experienced patients infected with HCV genotype 1 [11], [12].

However, the flexibility of the HCV genome, caused by the high error rate of its polymerase, allows the virus to adapt rapidly to the presence of an antiviral drug through the selection of minor variants with drug resistant mutations [13], [14]. Both clinical and replicon studies have demonstrated that resistant variants are characterized by mutations at positions V36, T54, R155 or A156 [15], [16]. Indeed, in 74% of patients who failed to respond to TVR combination treatment in phase 3 clinical TVR trials, the virus population was dominated by resistant variants immediately after treatment [Sullivan et al. Unpublished]. The abundant presence of resistant variants in patients who failed treatment is cause for concern, as this may limit the options for future retreatment in these patients and ultimately may also result in the spread of resistant viruses. Whether the virus population returns to baseline with respect to frequency of resistant variants is therefore an important issue to address. Using population and clonal sequencing, a few studies have monitored the frequency of resistant variants at different time points following TVR treatment in phase 1 and phase 3 clinical trials. These studies suggest that after termination of TVR treatment, the resistant virus population is gradually replaced by WT virus [17], [Sullivan et al. Unpublished]. The observed decline in frequency of resistant variants is not surprising as their fitness is impaired compared to WT virus [14], [15], [17].

The aim of the study presented here was to study the frequency of resistant variants in patients 4 years after 14-days of monotherapy with TVR using the novel ultra-deep pyrosequencing (UDPS) technique. The extreme sensitivity of UDPS enables a comparison of changes in frequency of minor variants compared to baseline far beyond the limit of detection of conventional techniques. In addition, the large number of sequences that are generated also allows for a robust statistical analysis of observed changes in the virus population.

## Materials and Methods

### Study Design and Patient Characteristics

The VX04-950-101 and VX05-950-103 clinical phase 1 studies investigated the safety and antiviral activity of TVR [9], [10]. Both studies were conducted at 2 collaborative sites in The Netherlands and one site in Germany in 2005 and 2006. These studies were conducted in full compliance with the guidelines of Good Clinical Practice and of the World Medical Assembly Declaration of Helsinki. Prior to study initiation, the protocol and informed consent form were reviewed and approved by the institutional review boards at each site. All patients provided written informed consent before participating in any study-related activity.

**Figure 1:**
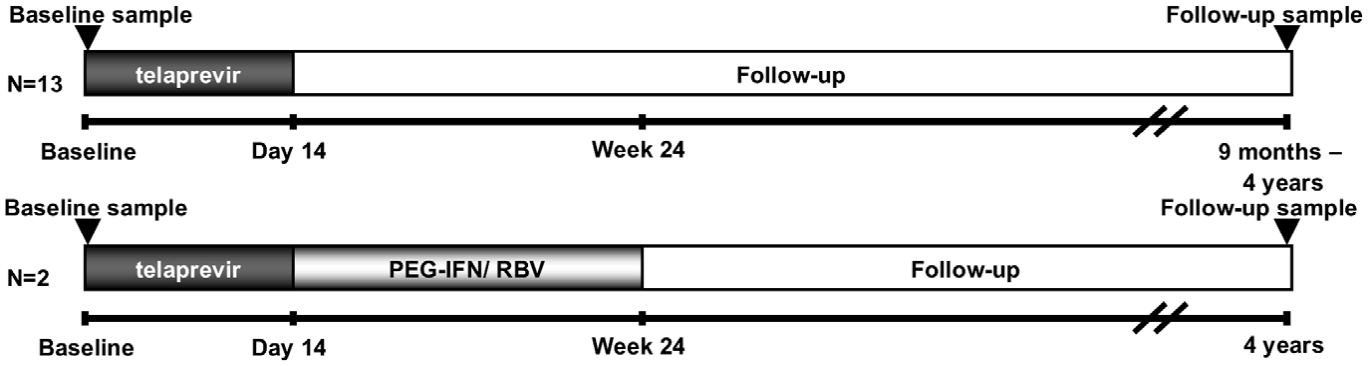
101 and 103 Study design. All 15 patients received TVR monotherapy for 14 days. Off-study treatment with PEG-IFN and RBV was offered to all study participants after day 14. Only 2 patients received the off-study standard of care.

For the 101-study, patients naïve or experienced to an interferon-based regimen were enrolled, whereas for the 103-study only treatment-naïve patients were eligible. All patients were chronically infected with HCV genotype 1. In the 101-study, 34 patients were randomized to receive placebo or TVR at doses of 450 mg or 750 mg every 8 hours or 1250 mg every 12 hours for 14 days. In the 103-study, 20 patients were randomized to receive TVR monotherapy, TVR with PEG-IFN or PEG-IFN with placebo for 14 days. At the completion of the 14-day study dosing, off-study standard of care with PEG-IFN and RBV was offered to all patients. The complete study-design is shown in Figure 1. During these studies, plasma samples for viral sequencing were collected at baseline, during, and after dosing.

In the study presented here, 15 patients from The Netherlands who received 14 days of TVR monotherapy in either the 101- or 103-study were included. Selection was based on availability of both a baseline and a long-term follow-up sample. A total of 12 and 3 patients were included from the 101- and 103-study, respectively. Patient characteristics including TVR-dosing are summarized in Table 1. Eight patients were infected with HCV genotype 1a and 7 patients with genotype 1b. Two patients (patient 14 and 15) subsequently received PEG-IFN and RBV, but failed to achieve a SVR. The interval between last TVR-dose and the follow-up time point ranged from 0.8 to 4.8 years, with a mean interval of 4 (±1.2) years. Mutations that were present at the end of treatment (EOT) at frequency ≥10% as determined by clonal sequencing are also summarized in Table 1 [15].

**Table 1:** Baseline characteristics of patients and previously published resistance mutations found at EOT.

Patient ID (age inyears, sex)	TVR dose (mg tid)	EOT resistance mutations^a^	Previous Treatment Outcome	GT	Baseline HCVRNA (IU/mL)	Follow-up HCVRNA (IU/mL)	Years between baseline and follow-up
1 (53, M)	450	V36M+R155K(75%)	NR	1a	1.0E+06	1.3E+06	2.0
2 (43, M)	450	V36M(20%),V36A(13%), R155K(16%), R155T(16%),R155I(11%), A156T(11%)	NR	1a	1.2E+06	3.2E+05	4.8
3 (47, M)	450	V36M(22%), V36A(13%), R155K(10%), R155T(18%)	N	1a	8.2E+06	6.7E+05	4.7
4 (52, M)	450	None ≥10%	NR	1b	2.6E+07	3.8E+06	3.4
5 (49, M)	450	T54A(22%), A156V(35%)	NR	1b	9.4E+06	2.2E+05	4.7
6 (33, M)	450	V36A(47%),T54A(22%), A156S(21%)	NR	1b	1.3E+06	1.5E+05	4.7
7^b^ (48, F)	750	T54A(26%)	NR	1b	5.6E+06	3.2E+06	4.6
8 (50, M)	750	V36M(25%),V36A(16%), R155K(26%), R155T(13%), R155I (16%)	NR	1a	9.2E+05	1.2E+06	4.5
9 (62, M)	1250	V36A(35%),T54A(27%), A156T(12%)	R	1b	2.2E+06	1.9E+06	1.9
10 (37, M)	1250	V36M(13%)	R	1a	3.1E+05	3.2E+05	4.5
11 (46, M)	1250	V36M(29%), V36M+R155K(49%)	N	1a	6.8E+06	1.8E+05	0.8
12 (44, M)	1250	T54A(67%), A156V(30%)	NR	1b	2.9E+06	2.3E+06	3.8
13 (52, F)	750	R155K(22%), V36M+R155K(63%), V36M+A156T(12%)	N	1a	1.1E+07	6.1E+05	3.9
14 (51, M)	750	A156V/T(100%)	N	1b	4.2E+06	4.9E+05	3.8
15 (61, M)	750	R155K(19%), V36M+R155K(57%)	N	1a	3.9E+07	4.8E+06	3.8

a Resistance mutations found at ≥10% of clonal population at EOT [15]. Viral variants were cloned at EOT and the proportion of resistant variants was calculated based on the total number of clones sequenced. ^b^End of treatment sequence data were not available for patient 7, short-term follow-up (7 to 10 days post dosing) data are provided here. GT,genotype; F, female; M, male, NR,non-responder; N,naïve; R,relapse.

### Genotyping and Viral Load

HCV RNA levels were determined using the Roche COBAS TaqMan HCV/HPS assay. Genotyping was performed according to Murphy et al.[18].

### HCV NS3 UDPS Sample Preparation, Sequencing and Mutation Analysis

HCV RNA for UDPS purposes was isolated from 100 µl plasma using the method described by Boom et al.[19]. Complementary DNA (cDNA) was synthesized from 9.4µl isolated RNA with the Transcriptor High Fidelity cDNA synthesis kit (Roche Applied Science). The amount of starting RNA was not normalized for each patient, as viral loads were all in the same range.

For UDPS, the 454 GS FLX titanium platform was used (Roche 454 Life Sciences, Branford, CT). The HCV NS3 protease region was amplified in 2 separate fragments. Forward primers comprised the 454 GS FLX titanium sequence primer A, followed by a patient-specific multiplex identifier sequence (MID) and a HCV-specific sequence. Reverse primers comprised the 454 GS FLX titanium sequence primer B followed by a patient-specific MID and a HCV-specific sequence as shown in Table 2.

**Table 2:** HCV NS3-specific UDPS-primers.

Genomic region and primer	Sequence	Position*	Amplicon coverage (bp)
**Amplicon NS3-I: V36/T54**			
VX1-Sense	5′- 454A-MID- AGCYTIACYGGCCGRGA - 3′	3477–3493	181
VX1-Antisense	5′- 454B-MID- TGGTCYACATTGGTRTACATYTG - 3′	3636–3658	
**Amplicon NS3-II: R155/A156**			
VX2-Sense	5′- 454A-MID- CAYGCYGATGTCATYCC -3′	3747–3763	298
VX2-Antisense	5′- 454B-MID- CCRCTICCIGTRGGRGC - 3′	4029–4045	

* Relative to H77 (GenBank accession number AF00960).

Amplicon NS3-I (251 bp, amino acid position 20–80) included the V36 and T54 positions and amplicon NS3-II (368 bp, amino acid position 109–208) included the R155 and A156 positions. Amplicons were generated for baseline and follow-up samples using the Expand High Fidelity^plus^ PCR system kit (Roche Applied Science). Amplicons were purified from agarose gels and quantified with the Quant-iT**™** dsDNA Assay Kit on a Qubit fluorometer (Invitrogen).

UDPS, preparation of library and analysis of amplicons was performed using the GS FLX titanium amplicon sequencing approach. Amplicons from baseline and follow-up were pooled separately. Sequencing was performed on a 2 region 454 GS FLX picotiterplate. Reads belonging to a specific patient and time point were separated based on the 454 GS FLX picotiterplate region and the patient-specific MID. The presence of variants at amino acid positions 36, 54, 155 and 156 was analyzed using the GS Amplicon Variant Analyzer (AVA) software version 2.0.01 from Roche. The AVA software performs quality control, trims primer derived sequences, maps the reads against a reference sequence and calculates the frequency of variants at designated positions. As reference sequence, the consensus NS3 protease sequence derived from the 15 included patients (separate for genotype 1a and 1b) was used.

### Sensitivity of UDPS

Using basic calculation of probabilities, the chance of detecting at least one specific minor variant (P[m]) with 95% certainty is: P[m] = 1-0.05 ^(1/N)^, where N is the number of individual reads. Thus, in theory, when analyzing 1000 reads, a minor variant present at 1-0.05^1/1000^ = 0.3% can be detected with 95% certainty.

### Analysis of Genetic Variability

To identify changes in quasispecies diversity, genetic variability was determined at both time points using average pairwise distances (APD) and average Shannon entropy (ASE) values. Genetic diversity of viral populations at both time points was determined by calculating the APD for each time point using a p-distance model [20] as implemented in MEGA 5.05 software [21]. The APD between baseline and follow-up were compared for both amplicons and separately for each genotype with a paired T-Test. The APD between genotypes were compared with the independent sample T-test.

The Shannon entropy was calculated with a script implemented in the software package ANDES [22]. The average Shannon entropy (ASE) per amplicon between time points and genotypes were also statistically compared using the same methods described for APD. In addition, Shannon entropy per positions of NS3-I and NS3-II were calculated for both time points.

### Statistical Analysis

Resistant variants were tabulated at each time point and compared statistically between time points at each position, evaluating the null hypothesis of equivalence between time points (baseline and follow-up) in the proportion of resistant variants. This was approached in 2 ways: (i) in an evaluation of the hypothesis of equivalence across all patients, and (ii) in an evaluation of the hypothesis of equivalence between time points independently for each patient and at each position.

(i) To test for the equivalence of the proportion of resistant variants between time points *across* patients, the arcsine-square root transformed percentage of resistant variants present at each time point was compared using least squares estimation with variation between subjects controlled by considering the ‘patient’ to be a random variable and considering ‘time point’ a fixed variable in a mixed model (JMP, v8.0.1). In this way, the effect of the fixed variable of time was tested while controlling for patient-level variation. The hypothesis of equivalence between time points in the proportion of resistant variants was evaluated independently for each position.

(ii) The count of resistant (V36A/M, T54A/S, R155K/T/M/I, and A156S/T/V) and ‘non-resistant’ variants was tabulated at each position independently for each patient. The null hypothesis of equivalence was evaluated independently at each position (4 positions; NS3-36, 54, 155, and 156) and for each patient (n = 20) using a two-sided Fisher’s Exact test (R, v.2.8.1), for a total of 80 tests of hypothesis (4×20 = 80). Type I error was controlled using a Bonferonni correction.

### Phylogenetic Analysis

For one patient (patient 15) NS3 clonal analysis of the EOT [17] and follow-up time point was performed. For clonal sequencing of the follow-up time point, viral RNA was extracted from plasma using the QIAamp BioRobot 9604 (Qiagen, Valencia, CA; Kit 965662). A cDNA fragment was synthesized from viral RNA and amplified by nested PCR. Agarose gel purified amplicons containing the entire NS3 protease coding region were cloned using the TOPO® XL PCR Cloning Kit (Invitrogen Corp). Cloning plates were sent to Beckman-Coulter (Agencourt® Biosciences; Danvers, MA), where 96 clones were amplified and sequenced.

For this patient the evolutionary history of resistant variants detected at follow-up was reconstructed through time using the Bayesian inference framework implemented in the program BEAST 1.6.1 [23]–[25]. Given an alignment of sequences sampled at different points in time, the rate of viral evolution and the phylogenetic history of infection were estimated on the observed time scale [26], [27]. Sequences were analysed using the Hasegawa, Kishino & Yano substitution model with gamma distribution under a relaxed molecular clock. A constant-size coalescent model was applied. Using this model structure, the dates of the most common recent ancestor (tMRCA) of the resistant variants present at follow-up were estimated using Bayesian Bayesian Markov Chain Monte Carlos (MCMC) sampling. The MCMC algorithm was run for 5*10^7^ states sampling every 5*10^3^ states. Seven independent runs were combined using Logcombiner v1.6.1. Chain convergence and posterior distributions were investigated using Tracer v1.5.

## Results

### UDPS Results

Amplification of fragments NS3-I and NS3-II succeeded in 13 out of 15 patients at both baseline and follow-up. For the 2 remaining patients (patient 9 and patient 10) amplification of fragment NS3-I failed at both baseline and follow-up. UDPS of fragment NS3-I succeeded in 12 out of 13 amplicons at both baseline and follow-up and in 14 out of 15 amplicons for fragment NS3-II. UDPS of one patient (patient 6) failed in both fragments at both baseline and follow-up.

UDPS of fragment NS3-I, spanning positions V36 and T54, resulted in a median of 14840 sequence reads (range 8687–20588) and 13717 sequence reads (range 8687–19968), per position in baseline samples. UDPS of fragment NS3-II, spanning positions R155 and A156, resulted in a median of 4017 (range 545–7363) and 3994 (range 545–7361) sequence reads, respectively, per position as shown in Tables 3 and 4.

**Table 3:** HCV-1a NS3 UDPS mutation analysis.

	HCV-1a Amplicon NS3-I								HCV-1a Amplicon NS3-II							
	V36				T54				R155				A156			
ID	Baseline		Follow-up		Baseline		Follow-up		Baseline		Follow-up		Baseline		Follow-up	
1	0	*(13817)*	0.05 M	*(10541)*	0	*(13113)*	0	*(9386)*	0	*(2977)*	0	*(4854)*	0	*(2977)*	0	*(4853)*
2	**0.28 L**; 0.04 A	*(12481)*	0	*(5383)*	0	*(12305)*	S 0.04	*(5327)*	0	*(545)*	0	*(2780)*	0	*(545)*	0	*(2757)*
3	0.03 A	*(18294)*	0	*(15074)*	0	*(17191)*	A 0.02	*(13650)*	0	*(3534)*	0	*(2249)*	0	*(3532)*	0	*(2248)*
8	0.08 L	*(16263)*	0	*(4791)*	0	*(14215)*	0	*(4517)*	0	*(5453)*	0	*(8700)*	0	*(4456)*	0	*(8696)*
10		*NA*		*NA*		*NA*		*NA*	0	*(5379)*	**0.55 G**	*(8154)*	0	*(5379)*	0	*(8146)*
11	0	*(8687)*	0	*(6448)*	0	*(8687)*	I 0.01	*(10631)*	**0.14 S**	*(2873)*	0	*(2986)*	0	*(2873*	0	*(8116)*
13	0	*(20588)*	0	*(17458)*	0	*(19968)*	0	*(17201)*	0	*(5398)*	0	*(7147)*	0	*(5396)*	0	*(7144)*
15	0	*(18993)*	**4.53 M**	*(12836)*	0	*(18320)*	**S 0.35**	*(11769)*	0	*(5291)*	0	*(9032)*	0	*(5722)*	0.01 T	*(9032)*

Percentage of UDPS reads with mutations. The parenthetical values indicate the number of reads analyzed. The bold numbers indicate variation present at a frequency above 0.1%. Underlined amino acids indicate resistant variants that were also observed by clonal sequencing at EOT.

**Table 4:** HCV-1b NS3 UDPS mutation analysis.

	HCV-1b Amplicon NS3-I								HCV-1b Amplicon NS3-II							
	V36				T54				R155				A156			
ID	Baseline		Follow-up		Baseline		Follow-up		Baseline		Follow-up		Baseline		Follow-up	
4	0.02 A	*(16899)*	0	*(7312)*	0	*(16564)*	0	*(7224)*	0.02 S	*(4500)*	0	*(2459)*	0.02 G	*(4523)*	0.04 D; 0.04 V	*(2459)*
5	**0.39 I**	*(15804)*	0	*(13911)*	0.02 I	*(15191)*	0	*(13333)*	0	*(7363)*	0	*(5005)*	0.04 T	*(7361)*	0	*(5004)*
7	0	*(10184)*	0	*(88567)*	0	*(10068)*	A 0.02	*(86402)*	0	*(3030)*	0.03 W	*(7924)*	0	*(3029)*	0.05 D	*(7916)*
9		*NA*		*NA*		*NA*		*NA*	0	*(988)*	0	*(3046)*	0	*(988)*	0	*(3045)*
12	0	*(10433)*	0.03 G	*(3927)*	**0.54 A**	*(10257)*	0	*(3869)*	**0.72 P**	*(2340)*	0	*(1253)*	**0.68 G**	*(2340)*	0	*(1253)*
14	0	*(13875)*	0	*(8714)*	0	*(13218)*	0	*(8129)*	0.03 W	*(7180)*	0	*(20478)*	0	*(7180)*	0	*(20466)*

Percentage of UDPS reads with mutations. The parenthetical values indicate the number of reads analyzed. The bold numbers indicate variation present at a frequency above 0.1%. Underlined amino acids indicate resistant variants that were also observed by clonal sequencing at EOT. UDPS of both fragments (NS3-I, NS3-II) failed for patient 6 at both baseline and follow-up.

In follow-up samples, UDPS of fragment NS3-I, resulted in a median of 9628 (range 3927–88567) and 10009 (range 3869–86402) sequence reads at positions V36 and T54 respectively. A median of 4930 reads (range 1253–20478) and 6074 reads (range 1253-20466) for positions R155 and A156 respectively were analyzed for fragment NS3-II.

### Frequency of Resistant Variants

**Figure 2:**
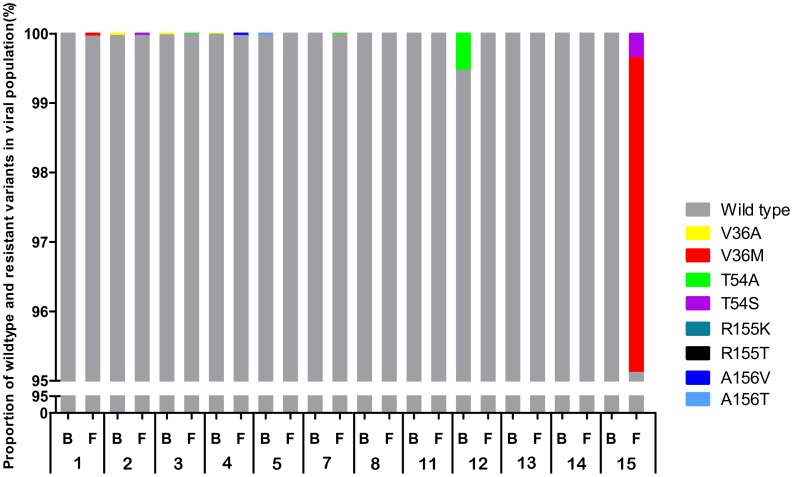
Prevalence of TVR resistance mutations at baseline and long-term follow-up. The percentage of resistant variants in the viral population at baseline (B) and at long-term follow-up time point (F) is depicted for each patient. For display purposes, only the segment from 95–100% is displayed; the portion of the viral population from 0–95% for all patients is WT. For comparison, viral population composition at the EOT time point is provided in Table 1. Note that amplification of amplicon NS3-I for UDPS failed for patients 9 and 10 (B, F). Furthermore UDPS of both fragments (NS3-I, NS3-II) failed for patient 6 (B, F).

Resistant variants, if present at either the baseline or follow-up time points, constituted a small fraction of the viral population (Figure 2, Table 3 and Table 4). Resistant variants were detected at baseline in 5 out of 12 patients. In 4 of these 5 patients, the baseline resistant variants were present at 0.04% of the population or less. In the remaining patient (patient 12) the baseline variant T54A was present at 0.54%. Together, resistant variants were detected at ∼0.5% or less of the viral population in 12 out of 14 patients.

As described before, at the end of the 14-day TVR-dosing period, all patients had TVR-selected variants as summarized in Table 1. After cessation of TVR-dosing, the proportion of the viral population comprising these TVR-selected variants decreased with a commensurate increase in the frequency of WT virus as published before [15], [17]. At the long-term follow-up assessment, the distribution of resistant variants was comparable to the baseline state. With the exception of 2 patients (patient 12 and 15; discussed below), resistant variants were detected at follow-up in 4 out of 11 patients with data from both amplicons, with a prevalence of 0.05% or less.

In one patient (patient 15) the combination of V36+T54S was observed in one variant. In all other patients no combination of resistance mutations was observed.

### Genetic Diversity at Baseline and Follow-up

**Figure 3:**
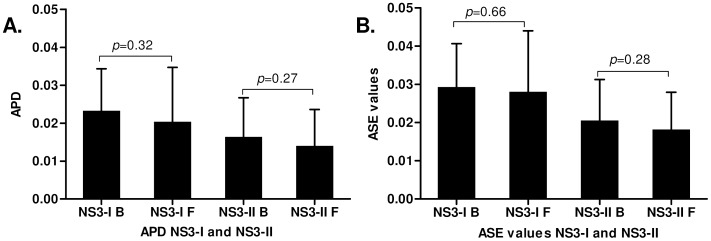
Genetic diversity at baseline and long-term follow-up. APD and ASE values are plotted in graph 3A and 3B respectively. No significant difference in the APD and ASE values were observed between baseline and follow-up. *P*-values are depicted in the graphs.

Both APD and ASE values were used to study changes in viral diversity between follow-up and baseline. No significant difference in the APD and ASE values were observed between the two time points as seen in Figure 3A and 3B. Subanalysis per genotype also did not indicate a differential diversity of the virus population between genotype 1a and 1b (data not shown).

**Figure 4:**
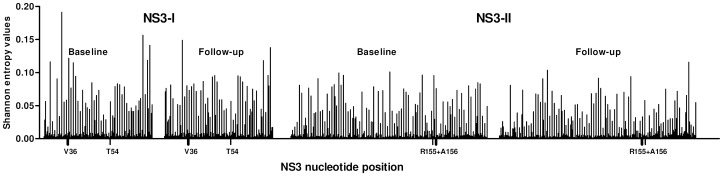
Shannon entropy per nucleotide position at baseline and follow-up. Shannon entropy per nucleotide position (averaged for all patients) is plotted for both amplicons and time points. Resistance associated amino acid positions are indicated on the X-axis.

In Figure 4 the Shannon entropy per position is plotted for both amplicons and time points averaged for all patients. No statistical significant difference was observed comparing baseline Shannon entropies to follow up.

### Test for Resistant Variant Enrichment: Baseline vs. Follow-up

The null-hypothesis of equivalent proportions of resistant variants between baseline and follow-up was evaluated independently at NS3 amino acid positions 36, 54, 155, and 156. Results suggested that, across patients, there was no difference in the frequency of resistant variants at baseline and follow-up. *P*-values were 0.53, 0.80, and 0.74 for NS3-36, 54, and 156. A comparison between time points in the case of NS3-155 variants was not possible, as no resistant variants were observed at this position at either time point (indicating equivalence between the time points).

To evaluate the same hypothesis individually (*i.e.,* to test for enrichment in the proportion of resistant variants independently for each patient), a Fisher Exact Test was performed for each patient and at each position. For 13 out of 14 patients, there was no suggestion of a significant difference between time points. For patient 12, there was a statistically greater prevalence of T54A at baseline (baseline; 0.54%) than at follow-up (0%; *p = *4.21e^-8^). This mutation was also found at the EOT time point in 67% of clones [15]. For patient 15, a statistically significant enrichment of both V36M and T54S (follow-up; 4.53% and 0.35%) variants at follow-up relative to baseline (both 0%; *p* =  <2.2e^-16^) was observed. Neither V36M nor T54S were detected at baseline in this patient but were present as 4.53% and 0.35% of the viral population at follow-up, respectively.

### Phylogenetic Analysis of Clonal Sequences of Patient 15

**Figure 5:**
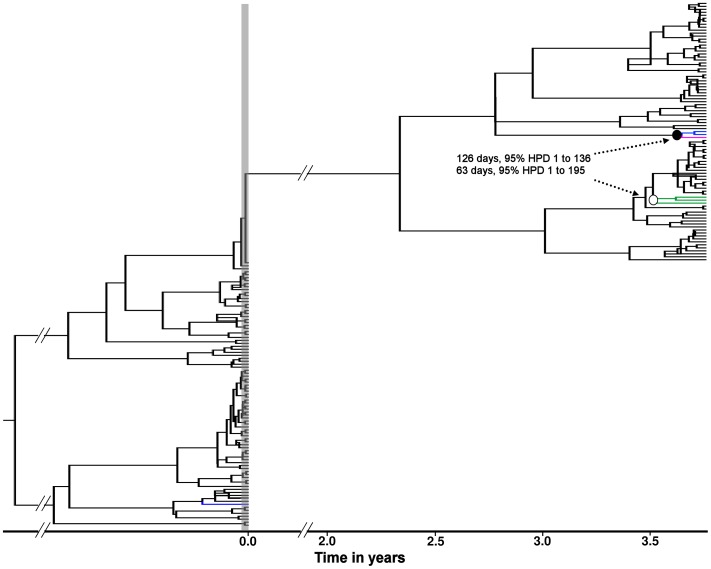
Molecular clock phylogeny from patient 15. The tree represents the maximum clade credibility tree from the Bayesian MCMC analysis. The bullets at the nodes indicate the tMRCA estimates for the V36M (• = 126 days, 95% HPD 1 - 360) and T54A/S (**○ = **63 days, 95% HPD 1 - 195) resistant variants. The V36M, T54A and T54S mutations are indicated in green, blue and pink respectively. The vertical grey shaded bar indicates the TVR-treatment period. Time scale in years relative to the EOT period is indicated on the X-axis.

At the EOT time point, the majority of clones sequenced from patient 15 were resistant, with V36M+R155K present in 57% of clones [17]. If the clones sequenced at the long-term follow-up time point had persisted for 4 years as a remnant of the resistant clones present immediately after dosing, it would be expected that in the phylogeny these resistant clones would cluster with the resistant clones present at the EOT. Instead, these clones formed part of the monophyletic clade that comprises the long-term follow-up viral population (Figure 5). In addition, calibrating the tree using BEAST, the MCMC analysis yielded tMRCA estimates for the resistant variants present at long term follow-up of 126 (95% highest posterior density (HPD) interval 1- 360) and 63 (95% HPD 1 -195) days for the V36M and T54A/S mutants respectively, suggesting de novo generation, rather than persistence, of the V36M and T54A/S clones observed at long-term follow-up.

## Discussion

In early phase 1 studies of TVR, it has been shown that resistant variants are rapidly selected in patients who received 14 days of TVR monotherapy. Assessment of the viral population 3-7 months after the end of TVR-dosing by clonal sequencing showed the predominance of WT virus in the majority of patients [17]. However, whether the viral population in these patients eventually returned to baseline state or whether resistant variants persisted at low levels is unknown.

This study was designed to investigate whether the selection of resistant variants after short-term TVR monotherapy results in long-term persistence of these variants. To address this question, a highly sensitive UDPS analysis of resistance was carried out on plasma samples taken after a median follow-up of 4 years after participation in the clinical phase 1 trial of 14-day TVR monotherapy [9], [10]. At this follow-up time point, frequency of resistance mutations was low and in general not increased compared to baseline frequencies. In addition, no significant overall change in quasispecies diversity, expressed by genetic distance or Shannon entropy, was present comparing the follow-up time point to baseline.

To our knowledge, this is the first study that investigated the frequency of protease inhibitor-resistant variants at baseline and after treatment using UDPS. Using UDPS, clonal and population sequencing, the sporadic presence of naturally occurring resistance mutations at low frequencies have been reported by others [17], [28]–[30]. In the present study, mutations associated with resistance were detected in baseline samples of naïve patients in 6 out of 12 patients, with frequencies of less than 0.1% in 4 of these 6 patients. Of note, the observed baseline resistant variants were not predictive of the presence of resistance at the end of the 14 days dosing period as in all but one patient resistant variants were detected at a level exceeding 10% post-dosing. Furthermore, the low level presence of resistance mutations at baseline did not necessarily result in selection of that variant during treatment as shown by the baseline A156T mutation in patient 5. Interestingly, mutations R155K or R155T, which are key mutations for resistance to both linear and macrocyclic protease inhibitors in genotype 1a, were not detected in any of the baseline or follow-up samples. At the follow-up time point, frequency of resistant variants was in general not increased.

Only patient 15 had a small but statistically significant increase in the low-level resistant variants, V36M and T54S. Interestingly, the T54S mutant, that was considered enriched in patient 15 relative to baseline, was not observed by clonal sequencing of 88 clones at EOT [15]. In addition, the phylogenetic analysis of clonal sequences of this patient suggests that the most recent common ancestors of the two clusters with resistant variants present at the follow-up time point are estimated to have an origin of 126 and 63 days before the long-term follow-up time point for the V36M and the T54S respectively. This suggests that in this patient the variants with the T54S and V36M mutations sequenced at follow-up are naturally occurring variants that arose after treatment.

Using conventional population or clonal sequencing others have reported a gradual decline to WT virus population after treatment discontinuation of either short term monotherapy or combination treatment with interferon for longer treatment periods [17], [31]. In other viral infections treated with DAAs, such as human immunodeficiency virus or hepatitis B virus infections, mechanisms to improve the fitness of resistant variants, such as selection of compensatory mutations, enable the resistant variants to persist. The short dosing period of 14 days was perhaps insufficient for the development of adaptive mutations that may restore fitness to WT levels. It is possible that with longer treatment durations, the fitness of resistant variants could be compensated by additional mutations that enhance the replication efficiency [17]. However, the evolution of resistant variants may be limited by the implementation of stopping rules that instruct to discontinue use of TVR in patients who are likely to have virologic failure. Furthermore, the currently approved TVR combination regimen includes PEG-IFN and RBV, which synergistically suppress virus replication thereby reducing the likelihood of occurrence and persistence of mutations.

There are some limitations to this study. First, there is a theoretical possibility of oversampling but as viral loads of all samples exceeded 10^5^ IU/ml (or 5*10^5^ copies/ml), viral RNA input was at least 10^4^ virus copies per test, demonstrating that redundancy or oversampling was not a problem in the UDPS test set up. Second, while at EOT resistant variants were detected in all patients [15], at follow-up the results from three patients (patient 6, 9 and 10) were missing due to unsuccessful amplification or UDPS. However it is unlikely that this has affected the conclusion of our study, as these sequence failures were random. Third, the intrinsic error rate of the UDPS technique may have caused some of the variability that was observed. A cut off of 0.1% or even 0.5% is often used for reliable detection of mutants based on plasmid controls [32]. If we would have implemented such a cut off in this study, observed variation at resistant sites would have been even less than the limited variation already present, as most of the observed variation at resistance associated sites was present at a level of less than 0.5%. In stead, sequencing errors in our system set up seem to occur at a much lower level than 0.5%. This can be inferred from the fact that observed variation at the resistance associated sites was very limited with 100% WT amino acid residues and nucleotide conservation in most samples, as shown in Tables 3 and 4. The little variation that was observed resulted in amino acid changes that have been described as polymorphisms of resistance associated mutations, indicating that these mutations do not result in non-viable virus and could well be true variation.

Results from a previous study by Susser et al. [31] who used clonal sequencing indicate that at long-term follow-up after initial TVR-monotherapy the majority of the viral population consisted of wild-type variants. Our study confirms and extends the results from this study as we demonstrate that in most patients, frequencies of resistant NS3 variants after 4 years of a 14 day monotherapy course measured by an extremely sensitive sequence analysis technique are comparable to baseline. Since HCV is not known to be archived, patients could potentially be retreated in the future with more expanded combination therapy regimens that still contain TVR or other protease inhibitors from the same class. Indeed, in a recent study, such quadruple combination regimens, consisting of PEG-IFN, RBV, a protease- and a polymerase inhibitor was very powerful [33]. However, re-treatment clinical trials are necessary to fully understand the implications of resistance.

## References

[1] World Health Organization (2011) Hepatitis C. Fact sheet No. 164. Revised June 2011. Available: http://www.who.int/mediacentre/factsheets/fs164/en/index.html. Accessed 2012 Jan 23.

[2] Terrault NA, Berenguer M (2006) Treating hepatitis C infection in liver transplant recipients. Liver Transpl 12: 1192–1204 10.1002/lt.20865 [doi]. 16868944 10.1002/lt.20865

[3] Foy E, Li K, Wang C, Sumpter R Jr, Ikeda M, et al (2003) Regulation of interferon regulatory factor-3 by the hepatitis C virus serine protease. Science 300 1145–1148 10.1126/science.1082604 [doi];1082604 [pii]. 12702807 10.1126/science.1082604

[4] Foster GR (2004) Past, present, and future hepatitis C treatments. Semin Liver Dis 24 (Suppl.2): 97–104. 10.1055/s-2004–832934 [doi]. 10.1055/s-2004-832934 15346252

[5] McHutchison JG, Bartenschlager R, Patel K, Pawlotsky JM (2006) The face of future hepatitis C antiviral drug development: recent biological and virologic advances and their translation to drug development and clinical practice. J Hepatol 44: 411–421. S0168–8278(05)00777-4 [pii];10.1016/j.jhep.2005.12.001 [doi]. 10.1016/j.jhep.2005.12.001 16364491

[6] Pawlotsky JM, McHutchison JG (2004) Hepatitis C. Development of new drugs and clinical trials: promises and pitfalls. Summary of an AASLD hepatitis single topic conference, Chicago, IL, February 27-March 1, 2003. Hepatology 39: 554–567. 10.1002/hep.20065 [doi]. 10.1002/hep.20065 14768012

[7] Bacon BR, Gordon SC, Lawitz E, Marcellin P, Vierling JM, et al (2011) Boceprevir for previously treated chronic HCV genotype 1 infection. N Engl J Med 364: 1207–1217. 10.1056/NEJMoa1009482 [doi]. 10.1056/NEJMoa1009482 PMC3153125 21449784

[8] Poordad F, McCone J Jr, Bacon BR, Bruno S, Manns MP, et al (2011) Boceprevir for untreated chronic HCV genotype 1 infection. N Engl J Med 364: 1195–1206. 10.1056/NEJMoa1010494 [doi]. 10.1056/NEJMoa1010494 PMC3766849 21449783

[9] Forestier N, Reesink HW, Weegink CJ, McNair L, Kieffer TL, et al (2007) Antiviral activity of telaprevir (VX-950) and peginterferon alfa-2a in patients with hepatitis C. Hepatology 46: 640–648. 10.1002/hep.21774 [doi]. 10.1002/hep.21774 17879366

[10] Reesink HW, Zeuzem S, Weegink CJ, Forestier N, van VA, et al (2006) Rapid decline of viral RNA in hepatitis C patients treated with VX-950: a phase Ib, placebo-controlled, randomized study. Gastroenterology 131: 997-1002. S0016-5085(06)01539-3 [pii];10.1053/j.gastro.2006.07.013 [doi]. 10.1053/j.gastro.2006.07.013 17030169

[11] Jacobson IM, McHutchison JG, Dusheiko G, Di Bisceglie AM, Reddy KR, et al (2011) Telaprevir for previously untreated chronic hepatitis C virus infection. N Engl J Med 364: 2405–2416. 10.1056/NEJMoa1012912 [doi]. 10.1056/NEJMoa1012912 21696307

[12] Zeuzem S, Andreone P, Pol S, Lawitz E, Diago M, et al (2011) Telaprevir for retreatment of HCV infection. N Engl J Med 364: 2417–2428. 10.1056/NEJMoa1013086 [doi]. 10.1056/NEJMoa1013086 21696308

[13] Bartenschlager R, Lohmann V (2000) Replication of hepatitis C virus. J Gen Virol 81: 1631–1648. 10859368 10.1099/0022-1317-81-7-1631

[14] Lin C, Lin K, Luong YP, Rao BG, Wei YY, et al (2004) In vitro resistance studies of hepatitis C virus serine protease inhibitors, VX-950 and BILN 2061: structural analysis indicates different resistance mechanisms. J Biol Chem 279: 17508–17514. 10.1074/jbc.M313020200 [doi];M313020200 [pii]. 10.1074/jbc.M313020200 14766754

[15] Kieffer TL, Sarrazin C, Miller JS, Welker MW, Forestier N, et al (2007) Telaprevir and pegylated interferon-alpha-2a inhibit wild-type and resistant genotype 1 hepatitis C virus replication in patients. Hepatology 46: 631–639. 10.1002/hep.21781 [doi]. 10.1002/hep.21781 17680654

[16] Sarrazin C, Zeuzem S (2010) Resistance to direct antiviral agents in patients with hepatitis C virus infection. Gastroenterology 138: 447–462. S0016-5085(09)02113-1 [pii];10.1053/j.gastro.2009.11.055 [doi]. 10.1053/j.gastro.2009.11.055 20006612

[17] Sarrazin C, Kieffer TL, Bartels D, Hanzelka B, Muh U, et al (2007) Dynamic hepatitis C virus genotypic and phenotypic changes in patients treated with the protease inhibitor telaprevir. Gastroenterology 132: 1767–1777. S0016-5085(07)00394-0 [pii];10.1053/j.gastro.2007.02.037 [doi]. 10.1053/j.gastro.2007.02.037 17484874

[18] Murphy DG, Willems B, Deschenes M, Hilzenrat N, Mousseau R, et al (2007) Use of sequence analysis of the NS5B region for routine genotyping of hepatitis C virus with reference to C/E1 and 5' untranslated region sequences. J Clin Microbiol 45: 1102-1112. JCM.02366-06 [pii];10.1128/JCM.02366-06 [doi]. 10.1128/JCM.02366-06 PMC1865836 17287328

[19] Boom R, Sol CJ, Salimans MM, Jansen CL, Wertheim-van Dillen PM, et al (1990) Rapid and simple method for purification of nucleic acids. J Clin Microbiol 28: 495–503. 1691208 10.1128/jcm.28.3.495-503.1990 PMC269651

[20] Nei M, Kumar S (2000) Molecular Evolution and Phylogenetics.New York: Oxford University Press.

[21] Tamura K, Peterson D, Peterson N, Stecher G, Nei M, et al (2011) MEGA5: molecular evolutionary genetics analysis using maximum likelihood, evolutionary distance, and maximum parsimony methods. Mol Biol Evol 28: 2731-2739. msr121 [pii];10.1093/molbev/msr121 [doi]. 10.1093/molbev/msr121 PMC3203626 21546353

[22] Li K, Venter E, Yooseph S, Stockwell TB, Eckerle LD, et al (2010) ANDES: Statistical tools for the ANalyses of DEep Sequencing. BMC Res Notes 3: 199. 1756-0500-3-199 [pii];10.1186/1756-0500-3-199 [doi]. 10.1186/1756-0500-3-199 PMC2921379 20633290

[23] Drummond AJ, Rambaut A, Shapiro B, Pybus OG (2005) Bayesian coalescent inference of past population dynamics from molecular sequences. Mol Biol Evol 22: 1185–1192. msi103 [pii];10.1093/molbev/msi103 [doi]. 10.1093/molbev/msi103 15703244

[24] Drummond AJ, Ho SY, Phillips MJ, Rambaut A (2006) Relaxed phylogenetics and dating with confidence. PLoS Biol 4: e88. 05-PLBI-RA-0392R4 [pii];10.1371/journal.pbio.0040088 [doi]. 10.1371/journal.pbio.0040088 PMC1395354 16683862

[25] Drummond AJ, Rambaut A (2007) BEAST: Bayesian evolutionary analysis by sampling trees. BMC Evol Biol 7: 214. 1471–2148–7-214 [pii];10.1186/1471-2148-7-214 [doi]. 10.1186/1471-2148-7-214 PMC2247476 17996036

[26] Strimmer K, Pybus OG (2001) Exploring the demographic history of DNA sequences using the generalized skyline plot. Mol Biol Evol 18: 2298–2305. 11719579 10.1093/oxfordjournals.molbev.a003776

[27] Pybus OG, Rambaut A, Harvey PH (2000) An integrated framework for the inference of viral population history from reconstructed genealogies. Genetics 155: 1429–1437. 10880500 10.1093/genetics/155.3.1429 PMC1461136

[28] Bartels DJ, Zhou Y, Zhang EZ, Marcial M, Byrn RA, et al (2008) Natural prevalence of hepatitis C virus variants with decreased sensitivity to NS3.4A protease inhibitors in treatment-naive subjects. J Infect Dis 198: 800–807. 10.1086/591141 [doi]. 10.1086/591141 18637752

[29] Kuntzen T, Timm J, Berical A, Lennon N, Berlin AM, et al (2008) Naturally occurring dominant resistance mutations to hepatitis C virus protease and polymerase inhibitors in treatment-naive patients. Hepatology 48: 1769–1778. 10.1002/hep.22549 [doi]. 10.1002/hep.22549 PMC2645896 19026009

[30] Nasu A, Marusawa H, Ueda Y, Nishijima N, Takahashi K, et al (2011) Genetic heterogeneity of hepatitis C virus in association with antiviral therapy determined by ultra-deep sequencing. PLoS One 6: e24907. 10.1371/journal.pone.0024907 [doi];PONE-D-11–11004 [pii]. 10.1371/journal.pone.0024907 PMC3178558 21966381

[31] Susser S, Vermehren J, Forestier N, Welker MW, Grigorian N, et al (2011) Analysis of long-term persistence of resistance mutations within the hepatitis C virus NS3 protease after treatment with telaprevir or boceprevir. J Clin Virol. S1386–6532(11)00342-8 [pii];10.1016/j.jcv.2011.08.015 [doi]. 10.1016/j.jcv.2011.08.015 21924672

[32] Tsibris AM, Korber B, Arnaout R, Russ C, Lo CC, et al (2009) Quantitative deep sequencing reveals dynamic HIV-1 escape and large population shifts during CCR5 antagonist therapy in vivo. PLoS One 4: e5683. 10.1371/journal.pone.0005683 [doi]. 10.1371/journal.pone.0005683 PMC2682648 19479085

[33] Lok AS, Gardiner DF, Lawitz E, Martorell C, Everson GT, et al (2012) Preliminary study of two antiviral agents for hepatitis C genotype 1. N Engl J Med 366: 216–224. 10.1056/NEJMoa1104430 [doi]. 10.1056/NEJMoa1104430 22256805

